# Interrogating the limits of precedent autonomy: the anomalous exclusion of basic care from the ambit of advance decisions

**DOI:** 10.1093/medlaw/fwaf030

**Published:** 2025-09-01

**Authors:** Samantha Halliday, Jean V McHale

**Affiliations:** Durham Law School, Durham University, Durham, DH1 3LE, United Kingdom; Birmingham Law School, University of Birmingham, Birmingham, B15 2TT, United Kingdom

**Keywords:** advance decision, autonomy, basic care, code of practice, human dignity, precedent autonomy

## Abstract

The Mental Capacity Act 2005 enables individuals to make advance decisions to refuse medical treatment once they lose mental capacity. However, scant attention has been given to the limit imposed by the Code of Practice upon the ability of an individual to refuse *care*, as opposed to *treatment* in an advance decision. This article examines the different meanings of ‘basic care’. It interrogates the genesis of the exclusion within the Code of Practice. The article examines the problems created by the exclusion, both in definitional terms and in relation to the conflict created with respect for precedent autonomy. It argues that while such an exclusion could be justified on the basis of public policy at the turn of the 21st century, the evolution of greater respect for patient-centred decision-making and respect for human dignity require its re-evaluation. The article challenges the continued relevance of the exception in the light of shifts in public policy and the case law, proposing that the ability to make an advance decision about basic care as well as treatment is an essential element in the toolkit designed to ensure individualized care at the end of life.

## I. INTRODUCTION

The tensions around determining to what extent an individual should be able to bind themselves via an advance decision before they lose mental capacity echo across the millennia from the legend of Odysseus to the present day.^1^ At the core of this tension lie the asymmetries between contemporaneous and anticipatory decisions, the idea that individuals must be protected from making unwise binding decisions that they will be unable to change due to intervening incapacity.^2^ It is well recognized in English law that an individual with capacity can refuse medical treatment, including life-sustaining treatment, and that such a refusal will bind healthcare professionals.^3^ That same individual may refuse care, including pain relief, food and fluids, even if that refusal is motivated by a desire to die. The Mental Capacity Act 2005 (MCA) recognises the right of the individual to refuse treatment in advance of incapacity.^4^ However, the Act's Code of Practice (COP) appears to prevent the same individual from refusing what might be termed 'basic care' in an advance decision.^5^ In this article, we argue that it is time to revisit the longstanding distinction between basic care and treatment to more effectively uphold precedent autonomy through advance decision making, and we outline a framework for how this can be achieved.

Scant attention has been given to the limitations imposed upon the ability of an individual to refuse medical *care*, as opposed to *treatment* in an advance decision. As we demonstrate below, this crucial distinction between care and treatment refusal is well-accepted in the clinical and policy literature, but lacks a legal basis in either statute or common law. Nevertheless, the limitation imposes a significant restriction upon the exercise of precedent autonomy in the name of what can best be described as ill-defined public policy considerations; a restriction that is inconsistent with the protection and promotion of precedent autonomy and the ethos underpinning the MCA that decisions should be taken by those they affect wherever possible.

The MCA provides that advance decisions have normative force, but contemporaneous and anticipatory decisions are not treated in the same way; precedent autonomy is subject to restrictions in terms of both the permissible content and form adopted by advance decisions.^6^ Although there is nothing in the legislation itself to suggest that basic care cannot be refused in an advance decision, the Code of Practice to the MCA excludes ‘basic or essential care’^7^ from the ambit of a valid advance refusal.^8^ As we discuss below,^9^ the Code of Practice is soft law; it is not legally binding, but there is an expectation of compliance by those to whom the Code is addressed. Given the widespread acceptance of this exclusion by healthcare professionals and professional bodies, we argue that the Code has introduced a significant limitation upon the exercise of precedent autonomy. The restriction remains in the draft revised Code published in 2022,^10^ despite the fact that no such limitation is imposed upon contemporaneous decision-making, diminishing respect for autonomy in the context of anticipatory decisions. As we argue below, even if such an exclusion were justifiable in 2005 when the Act was passed, it is inconsistent with the evolution of the case law in this area.

The definition of basic care itself is contested, and we suggest that both its exclusion and its fluid nature undermine the philosophy underpinning the MCA to empower individuals to take decisions for themselves wherever possible. Since the MCA came into force, three fundamental shifts in the case law can be observed: the increased prominence given to respect for human dignity;^11^ the adoption of a more patient-centred focus in the determination of an individual’s best interests;^12^ and that generally greater weight is being attributed to the known wishes of patients who lack capacity.^13^ Each of these shifts represents a considerable change from the prevailing attitudes in the 1990s when the Law Commission was undertaking its work in this area, which formed the basis of the Act.^14^ This raises the question of whether it is appropriate and indeed justifiable to continue to impose restrictions upon the scope of advance refusals of medical treatment, given the very different legal context in which healthcare professionals now practice and decisions are made for those who lack capacity.

This article interrogates the genesis of the exclusion of basic care from the ambit of a valid advance decision, arguing that while such an exclusion could be justified on the basis of public policy at the turn of the 21st century, the significant shifts that have occurred in public policy towards patient-centred decision-making and respect for human dignity require its re-evaluation. We fill a gap in the existing literature by analysing the discrepancy between the significance generally attributed to advance refusals of medical treatment in comparison to the approach taken to the same individual’s wishes and feelings relating to what might be termed basic, or nursing care. The significance lies in highlighting and examining the problems created by the exclusion, both in definitional terms and in relation to the conflict created with respect for precedent autonomy and human dignity. We challenge the continuing relevance of the exception in the light of shifts in public policy and the case law, proposing that the ability to make an advance decision about care as well as treatment is an essential element in the toolkit designed to ensure individualized care at the end of life.

## II. WHAT IS BASIC CARE?

Although the Code purports to render the advance refusal of ‘basic care’ invalid, there is no single definition of ‘basic care’; its scope remains one of the most controversial and emotive topics. This is particularly clear in two contexts: the provision of pain relief^15^ and that of nutrition and hydration.^16^ In the context of advance decisions, whether something falls within the category of care or medical treatment is highly significant.^17^ If the refusal relates to ‘basic care’, it is purportedly excluded by the Code; if it constitutes a refusal of medical treatment, it will bind healthcare professionals provided that it is otherwise valid and applicable under the MCA.

Taken at its broadest, basic care can include regular turning of patients in their hospital beds (to avoid pressure sores), washing/hygiene measures, feeding and hydration, pain relief, the provision of warmth and shelter, and management of symptoms such as vomiting and breathlessness. None of these activities could be considered ‘physical contact which is generally acceptable in the ordinary conduct of daily life’^18^ and therefore each would normally require consent. Where the individual concerned has capacity, that consent may well be implied, for example by holding out an arm for washing; where the individual lacks capacity, such care can be provided under the auspices of section 5 MCA (the general legal authority), provided that the person providing the care reasonably believes that it will be in their best interests for the care to be provided.

Basic care can be seen as activity intended to protect the dignity of the patient, encompassing the minimum level of care that all people should be afforded so that they are washed, clothed, fed, and kept warm. In the context of end of life care, basic care itself may constitute part of a managed death. Withholding of such care can be framed as neglect or abandonment of the patient,^19^ regardless of whether or not the patient is aware of the care provided. It has also been argued that basic care must be provided in order to safeguard the dignity and sensibilities of those caring for the patient and other patients. We suggest that it is highly questionable whether the provision of basic care in the face of an advance refusal drafted whilst the patient had capacity, can be considered protective of their dignity, or of human dignity in general. Moreover, we argue that basic care choices should focus upon the individual and not be determined by the interests of others. As discussed below, there are multiple definitions of dignity and in the case of basic care a construction of dignity as constraint (emphasizing the interests of the community of which P is a part) appears to be being adopted, rather than an individualistic construction that promotes dignity as empowerment, reinforcing the individual’s right to make decisions for themselves.^20^ This communitarian construction of dignity and the diminished importance accorded to autonomy is out of line with public policy in the 21st century and is interrogated further below.

Significantly, the designation of an act as ‘basic care’ does not mean that it is unimportant; rather, as Denise Dudzinski and Sarah Shannon note, basic care, sometimes referred to as nursing care, can be life-sustaining. They argue:

Cleaning patients after urinary or faecal incontinence prevents skin breakdown and reduces the risk of infection. Turning patients who are bedridden is a crucial component of maintaining skin integrity to prevent skin breakdown or bedsores. ^21^

As they note, withdrawal of basic care can lead to ‘life-threatening complications’ that ultimately can precipitate death.^22^ It has been suggested that a ‘web of expectation’ operates;^23^ that ‘patients’ are expected to consent to basic nursing care, such as feeding and hygiene measures. Nevertheless, where the individual has capacity, if their refusal of consent is ignored, the touching would constitute an unlawful trespass to the person. Why then are anticipatory refusals of basic care treated as non-binding, and on what basis can the differential treatment of anticipatory and contemporaneous refusals be justified?

## III. THE EVOLUTION OF THE BASIC CARE EXCEPTION IN THE CODE OF PRACTICE

### A. The Law Commission consultation

To fully understand the approach to basic care today in terms of the interpretation of the MCA Code and its impact on patients’ advance decisions, it is necessary to examine the background to the development of the legislation. The Law Commission undertook an extensive review of the law concerning mental capacity (1989–1995).^24^ Its proposals included a recommendation that legislative provision be made for advance decisions.^25^

Judicial support affirming the need for respect for precedent autonomy can be traced back to the early 1990s, providing a backdrop to the Law Commission’s deliberations. As Lord Donaldson memorably stated in *Re* T^26^

Prima facie every adult has the right and capacity to decide whether or not he will accept medical treatment, even if a refusal may risk permanent injury to his health or even lead to premature death. Furthermore, it matters not whether the reasons for the refusal were rational or irrational, unknown or even non-existent. This is so notwithstanding the very strong public interest in preserving the life and health of all citizens.^27^

Lord Donaldson’s approach prioritizing self-determination was followed in *Airedale NHS Trust v Bland*.^28^ Lord Goff recognized that a patient with mental capacity could refuse ‘To consent to treatment or care by which his life would or might be prolonged, the doctors responsible for his care must give effect to his wishes, even though they do not consider it to be in his best interests to do so.’^29^ He went further than Lord Donaldon in *Re T*, stressing that it is not only ‘the principle of the sanctity of human life [that] must yield to the principle of self-determination … [but], for present purposes perhaps more important, the doctor’s duty to act in the best interests of his patient must likewise be qualified’.^30^ Crucially, he then went on to state that this also applied in situations where a patient had refused treatment prior to lacking capacity or becoming unable to communicate a decision.^31^ Nevertheless, despite judicial support for advance decisions, the British Medical Association took the view that whilst they were important, they should be regarded as influential, rather than necessarily binding upon doctors.^32^

The Law Commission’s Consultation document in 1993 recognized the existing legal uncertainty about the status of advance decisions and proposed that they should be placed on a statutory footing.^33^ However, it suggested that the ability to refuse treatment in advance of incapacity should not be absolute. This approach was influenced by arguments made by Andrew Grubb in a 1993 *Medical Law Review* case commentary on the decision in *Re T*.^34^ In that case, Lord Donaldson emphasized that an adult with capacity could refuse any treatment, including life-sustaining treatment, but had continued (*obiter*) to state that an exception might be applicable in the case of a pregnant person where their choice ‘may lead to the death of a viable foetus.’^35^ Grubb engaged with this potential reservation but suggested that the exception was actually much broader than the context of pregnancy and that a distinction should be drawn between care and treatment. Echoing the concerns about abandonment raised by Lord Hoffmann in *Bland*,^36^ Grubb argued that:

… a doctor cannot be required to refrain from treating a patient so that, in effect, the patient is abandoned. A patient *probably*, therefore, could not validly refuse basic care in the form of what is often termed ‘nursing care’. Public policy would prohibit a patient from refusing such treatment.^37^

Whilst the first sentence echoes Lord Hoffmann’s suggestion that there is a duty to provide basic care, Grubb goes further, suggesting that an individual cannot refuse basic care; indeed, he appears to argue that even contemporaneous refusals of basic care made by an individual with capacity would be ineffective.^38^ Grubb then went on to state that

Finally, the issue arises as to whether the interests of others can affect the patient’s right to refuse treatment. The administration of ‘nursing care’ may be such an example, based upon the interests of the health care professionals and other patients who may be affected by a patient who refuses nursing care. A more controversial example would be the pregnant woman who refuses life-sustaining treatment.^39^

In its 1993 Consultation Document, the Law Commission cited Grubb, but went further. Despite endorsing a model of decision-making designed to empower patients to make decisions for themselves, the Law Commission proposed that ‘An anticipatory decision should be regarded as ineffective to the extent that it purports to refuse pain relief or basic care including nursing care or spoon feeding.’^40^ Thus, it distinguished both basic care and pain relief, concluding that they should be provided in accordance with ‘the patient’s current needs and wishes (if his wishes can be discerned) *without reference to his prior instructions.*’^41^ That same year, commenting on the House of Lords’ decision in *Bland* in 1993, Kennedy and Grubb examined spoon feeding and analysed whether the mode of delivery of nutrition and hydration could form the basis of a distinction between ‘treatment’ and ‘care’.^42^ They rejected such a distinction, echoing Sir Thomas Bingham MR^43^ in arguing that it was not a matter of labels, but rather that a doctor’s duty is to act in their patient’s best interests and, where the patient’s best interests lie in being allowed to die, that duty will require that the patient not be spoon-fed as that would frustrate the patient’s best interests.^44^ Nevertheless, they emphasized that ‘the doctor’s general duty to comfort would require the doctor to administer whatever medicine may be appropriate to alleviate any distress which may otherwise be caused to the patient.’^45^ Thus, Kennedy and Grubb did not consider that a doctor would inevitably have a duty to feed a patient, but they did not address the question of whether an individual could refuse spoon-feeding in an advance decision, a crucial distinction drawn by the Law Commission.^46^

### B. The Law Commission Mental Incapacity Report 1995

The Law Commission’s *Mental Incapacity Report* set out wide-ranging proposals for legislation concerning the care and treatment of adults lacking mental capacity,^47^ including provisions enabling adults with mental capacity to create legally binding advance decisions to refuse treatment. However, it concluded that public policy should limit the exercise of precedent autonomy due to its impact upon staff and on other patients,^48^ recommending that ‘An advance refusal of treatment should not preclude the provision of "basic care", namely care to maintain bodily cleanliness and to alleviate severe pain as well as the provision of direct oral nutrition or hydration.’^49^

Significantly, pain relief was included within the Report’s definition of basic care. However, the Law Commission accepted that ‘patients with capacity regularly refuse certain types or levels of pain relief because they prefer to maintain alertness’^50^ and therefore included only the ‘alleviation of severe pain’^51^ within the basic care that could not be excluded in advance. What amounts to ‘severe pain’ was not defined. Moreover, the Law Commission maintained the distinction between clinically assisted nutrition and hydration and what it now preferred to call ‘direct oral feeding to cater for the administration of nutrition or hydration by syringe or cup.’^52^ The Report stated, ‘Our proposed definition of "basic care" reflects a level of care which it would be contrary to public policy to withhold from a patient without capacity.’^53^ However, whilst the requirement that basic care be provided is grounded in a public policy justification, the Report does not specify which precise public policy justification the Law Commission supported.

### C. The slow move to legislation

Following the election of the Labour Government in 1997, the Law Commission’s proposals were set out for consultation in the document *Who Decides?*^54^ The Government sought views on the Law Commission’s recommendation that advance refusals should exclude ‘basic care’,^55^ expressing some concern that this might result in forced oral feeding.^56^ However, the subsequent proposals for making decisions on behalf of adults lacking capacity set out in *Making Decisions*, 1999,^57^ entirely omitted provision for advance decisions.^58^

This omission reflected opposition to advance decisions, often characterized by opponents as introducing euthanasia through the back door.^59^ This issue was particularly controversial in 1999. Firstly, the BMA guidance *Withholding and Withdrawing Life-prolonging Medical Treatment* (June 1999) emphasized the right of patients to refuse treatment both contemporaneously and in advance of incapacity, including nutrition and hydration, regardless of whether the patient was terminally ill or in a PVS. This document proved controversial, for example, the Times newspaper described it as a ‘Death Code’ and noted the creation of the ‘Medical Ethics Alliance’, a group of doctors opposed to the guidance.^60^ Secondly, concerns had also been raised that elderly patients were being allowed to starve to death.^61^ At the time of publication of *Making Decisions*, the Times newspaper reported that there were 60 cases being investigated by the police, more than half of which involved the decision to remove hydration and nutrition from elderly patients who were not terminally ill.^62^ Thirdly, public trust in doctors was seriously undermined by the investigation and conviction of the General Practitioner and serial killer Dr Harold Shipman for the murder of 15 patients.^63^ Shipman’s trial started in October 1999, the same month that *Making Decisions* was published. Against this backdrop of controversy, the Government argued that a combination of case law and the BMA’s Code of Practice^64^ would provide ‘sufficient clarity and flexibility to enable the validity and applicability of advance statements to be decided on a case-by-case basis.’^65^

Ultimately, the Government changed its mind, forming the view that advance decisions needed to be incorporated within the statutory framework.^66^ The provisions relating to advance decisions in the Draft Mental Incapacity Bill 2003,^67^ drew upon the Law Commission’s 1995 proposals, with the notable exception of a reference to a basic care exclusion. Despite its omission, the Joint Committee scrutinizing the Draft Bill devoted significant attention to the refusal of basic care and especially to the question of whether both CANH and oral feeding constitute medical treatment and thus would be capable of being refused in a valid and applicable advance decision.^68^ However, despite repeated questioning, witnesses giving evidence to the Joint Committee consistently confirmed that oral feeding is distinct from CANH, and that, as basic care, it should always be provided to patients.^69^ Moreover Dr Wilks, the then Chair of the BMA’s Medical Ethics Committee,^70^ stressed the need for a basic care exclusion, saying that this was not simply a matter of patients’ rights, but also those of healthcare professionals: ‘[it] would be very, very difficult for doctors to respect [advance refusals of care], partly for other health reasons in terms of other people and partly because doctors and nurses simply cannot leave somebody without those basic functions.’^71^

The Catholic Bishops’ Conference of England and Wales and the Linacre Centre for Healthcare Ethics argued that the definition of basic care should be broadened to include the continuation of ‘ordinary care already being given’, for example, insulin and the continuance of CANH through an established feeding tube.^72^ There was no consensus on the parameters of basic care, with definitions ranging from including oral feeding through to long-term medication. Ultimately, the Joint Committee concluded that to exclude advance decisions from the legislation would undermine the Bill’s intention ‘to create a comprehensive and accessible framework of statutory legislation.’^73^ Nevertheless, it recommended that

The Bill should seek to draw a distinction between basic care (which would include the giving of nutrition and hydration by normal means as well as actions to assist general hygiene and comfort), and the use of artificial means of nutrition and hydration, such as drips or naso-gastric tubes.^74^

The latter would constitute treatment, while the former would fall outside it. Responding to the Report, the Government agreed that such a distinction should be drawn but noted that it would be ‘difficult to produce a statutory definition which commands universal agreement’ and consequently proposed to leave guidance concerning the scope of basic care to be detailed in the Code of Practice.^75^ This approach ultimately prevailed in the MCA.

## IV. THE LEGAL AND POLICY FRAMEWORK

### A. The Mental Capacity Act 2005

Largely in keeping with the Law Commission’s proposals,^76^ the MCA effectively codified the existing common law, recognizing that an individual can make a valid and binding advance refusal of treatment, including life-sustaining treatment, that will take effect if they later lack the capacity to make a contemporaneous decision.^77^ However, it imposed significant limitations upon the scope of anticipatory decision-making, restricting the ability to make advance decisions to those aged 18 and above and excluding the potential for advance consent to treatment.^78^ The validity of an advance refusal of treatment is dependent upon the individual being deemed to have the capacity to make the relevant treatment decision at the time the advance decision was made.^79^ In order to be effective, the advance decision must specify the particular treatment to be refused and the circumstances in which that refusal is to operate.^80^ Additional requirements are imposed upon advance refusals of life-sustaining treatment. These must be in writing, signed by the individual, or someone acting on their behalf in their presence, and witnessed, and include a specific statement that the refusal is intended to operate even if life is at risk.^81^

Provided that the individual’s advance decision is valid and applicable to the treatment in question, the decision will have ‘effect as if he had made it, and had had capacity to make it, at the time when the question arises whether the treatment should be carried out or continued’.^82^ However, even if an advance refusal satisfies the eligibility criteria, the continued validity and applicability of the advance refusal must be considered. An advance decision can be revoked at any time whilst P has the capacity to do so.^83^ Additionally, three categories of intervening events may invalidate the advance decision—where the individual subsequently creates a lasting power of attorney and devolves the authority to the donee to consent or refuse consent to the treatment covered by the advance decision^84^ where the individual has acted inconsistently with that decision since making it^85^ or where reasonable grounds exist for believing that circumstances exist that the individual had not anticipated, but which would have affected their decision.^86^

The permissible scope of advance decisions is limited to refusals of treatment; neither a request for treatment^87^ nor a request for assistance in dying^88^ will be binding, although treatment preferences expressed in an advance decision will fall to be considered under the assessment of best interests, as expressions of the person’s past wishes.^89^ Thus, just as in the contemporaneous context, the right to self-determination in the anticipatory context is conceived of as a right to decide not to be treated, a right to defend one’s bodily integrity, but not a right to demand treatment.^90^ However, a notable difference appears to exist in relation to the right to decide not to receive basic care.

### B. The MCA code of practice

Throughout the final legislative process, the government resisted calls to exclude basic care or to define what might constitute basic care on the face of the Act, preferring to leave this matter to the Code.^91^ At first sight, this could be seen as a pragmatic approach recognizing that it would be extremely difficult to attain consensus upon the definition of basic care, and the operation of that definition. Nonetheless, this exclusion has created a very problematic practical legacy, such a restriction upon the exercise of precedent autonomy has the potential for broad effect and renders the rights and duties of those concerned (patients and healthcare professionals alike) unclear. A key question is what status does the Code have, is it mere guidance, or does it represent backdoor regulation?

Section 42(5) MCA requires a court or tribunal conducting criminal or civil proceedings to take account of the Code’s provisions, or a failure to comply with the Code, where relevant to a question arising in the proceedings. Writing the foreword to the Code, Lord Falconer describes it as practical guidance, setting out how the Act will operate and providing examples of best practice.^92^ Similarly, the introduction recognizes that some people (including healthcare professionals caring for a person lacking capacity) have a legal duty to *have regard* to the Code, but clarifies that ‘The Act does not impose a legal duty on anyone to "comply" with the Code—it should be viewed as guidance rather than instruction.’^93^ According to the introduction, having regard to the Code merely requires that individuals acting, or making a decision on behalf of another person who lacks capacity, are aware of the Code and that, in the case of non-compliance, they will be expected to give good reasons for departure from it.^94^

In *An NHS Trust v Y*^95^, the Supreme Court emphasized that the MCA Code was simply guidance, rather than a source of obligation. The Code states that ‘The Court of Protection *must* be asked to make decisions relating to the proposed withholding or withdrawal of artificial nutrition and hydration (ANH) from a patient in a permanent vegetative state (PVS)’,^96^ but no corresponding duty is set out in the MCA. Lady Black stressed that ‘Whatever the weight given to the Code by section 42 of the MCA, it does not create an obligation as a matter of law to apply to court in every case.’^97^ Instead, she concluded that the Code ‘contains valuable guidance’, guidance to which those making decisions for individuals lacking capacity should have regard, but that it is not law.^98^ Nonetheless, subsequent judicial dicta have stressed the need for compliance with both the Code and the statutory provisions in preparing an advance decision.^99^

Thus, the Code is a form of soft law, it is not merely advisory; there is at least an expectation that the Code will be followed, or that deviation therefrom will be subject to justification. What is notable is its terminology around basic care. While it may have been expected that, as guidance, the Code would have utilized words such as *should*, the Code is phrased in exclusionary terms—an advance decision *cannot* refuse basic care. Paragraph 9.28 is not normative in nature, rather it appears that under the Code, there is no scope for evaluation or discretion to treat the individual’s advance decision as determinative of care to be provided. In this way, the Code purports to limit the Act.

A further complication is created by paragraph 9.28, which provides that healthcare professionals can provide basic care to the individual who lacks capacity under the general legal authority (section 5 MCA), provided that they reasonably believe it to be in P’s best interests. This will require them to take account of P’s ‘past and present wishes and feelings (and, in particular, any relevant written statement made by him when he had capacity).’^100^ However, given the Code’s declaration that an advance decision cannot refuse basic care, it would appear unlikely that P’s wishes will be deemed relevant in this context, despite the shift towards patient-centred care in practice.

Moreover, the general authority requires that P is treated in line with their best interests, but as we explain below, the exclusion of basic care from the ambit of an advance decision does not reflect the interests of P, instead it is an exclusion that is based upon the interests of others, of healthcare professionals, other patients and P’s own family and friends. In so doing, the Code recognizes diverse interests but prioritizes those of the people able to communicate them now. This restriction imposes a significant limitation upon the individual’s autonomy, promoting the welfare and interests of others at the expense of respect for the incapacitous individual’s precedent autonomy and respect for human dignity.

### C. Professional guidance and end of life policies

A further challenge is the role of professional guidance. In the years since the Code came into operation in 2007, there appears to have been no attempt to challenge the basic care exclusion; if anything, more recent guidance seems to expand its scope. One reason for this may be the impact of the ‘Liverpool Care Pathway’ (LCP).^101^ A protocol setting out the care pathway at the end of life for those dying in hospital, the LCP came under very great criticism and was the subject of an independent review in 2013, which noted many instances of serious concern and found that reports of poor treatment on the pathway abound.^102^ The inclusion of the withdrawal of CANH was not innovative, it was evidence-based practice, but the very name ‘pathway’ emphasized that this was a one-size-fits-all programme, that it was not designed to treat people as individuals. The setting in which the pathway was employed, in busy hospitals, rather than hospices, meant that often there was little time for discussion about what the patient wanted/would want. The Review found that:

There was a feeling that the drugs were being used as a ‘chemical cosh’ which diminished the patient’s desire or ability to accept food or drink. The apparently unnecessary withholding or prohibition of oral fluids seemed to cause the greatest concern.^103^

The LCP was phased out following the Report, replaced in 2015 by the NICE guidance *Care of dying adults in the last days of life*, which emphasizes the importance of individualized care, taking account of the individual’s wishes (including advance decisions) and promoting dialogue between the clinicians, the individual and those important to them.^104^ Nevertheless, the report’s focus upon the issues of pain relief and oral nutrition and hydration provides clear evidence of the uncertain boundaries of the term ‘basic care’.

In *Y*, Lady Black noted the importance of compliance with both the Code and professional guidance, noting the latter’s important role ‘in ensuring the proper protection of patients and in maintaining the confidence of the public in the health care system.’^105^ However, whilst there are overlaps, there are significant differences between guidance from the various professional organizations, that produced by the Department of Health, and the MCA Code.^106^ Moreover, there is no consensus upon what will constitute basic care and there is a proliferation of these ‘standards’ about end of life care.

The latest guidance issued by the GMC and BMA takes the position that basic care should not be refused through an advance decision.^107^ Significantly, although the BMA Core Ethics guidance suggests that there are limits to what may be validly refused in an advance decision, it does distinguish between the exclusion of advance requests for non-clinically indicated treatment (a limitation recognised by the Court of Appeal in *Burke*) and their own view that the refusal of basic care would be inappropriate.^108^ This could be seen as an acknowledgment of the fact that the exclusion of basic care set out in the MCA COP is not a limitation imposed by law. Both organizations have adopted a similar definition of basic care to that set out in the Code, although the Code goes further by including warmth and shelter in its examples of what will constitute basic care. In contrast, the professional guidance is limited to care which is likely to be provided by healthcare professionals, in each case listing hygiene, the offer of food and drink, and pain relief and, in the case of the BMA guidance, includes management of distressing symptoms such as breathlessness and vomiting.^109^ This latter example demonstrates that basic care goes beyond what might be considered nursing care as it anticipates the use of medication for symptom management; it is difficult not to regard this as treatment, the very form of care that may be refused by an advance decision.

Concern that patients might be denied access, or not be assisted to take in food and fluids, dominated the parliamentary debates during the passage of the Mental Capacity Act; it also formed the focus of both the Mid Staffordshire Inquiry^110^ and the Liverpool Care Pathway Inquiry.^111^ However, in each case, the focus was upon patients being deprived of nutrition and hydration, rather than upon their refusal thereof. These are very different situations; the first will constitute neglect,^112^ while the latter can be seen as an autonomous decision to stop eating and drinking. Nevertheless, the Code states that basic care will include ‘the offer of food and water by mouth,’^113^ a stance echoed by professional guidance.^114^ By excluding advance refusals of food and fluids, a clear limit is being placed upon precedent autonomy, a limitation that does not apply to those who retain capacity, including those who opt to voluntarily stop eating and drinking in order to bring about their own death.^115^ Thus, it is suggested that the code and professional standards serve to limit the exercise of precedent autonomy, a limit that has seemingly been adopted without detailed analysis of whether it is justifiable.

## V. INTERROGATING THE PUBLIC POLICY UNDERPINNING THE BASIC CARE EXCEPTION

In this section, we interrogate the theoretical basis for a basic care exception and its problematic nature. First, we consider the argument that allowing individuals to refuse basic care can be seen as incompatible with respect for human dignity. Secondly, we critique Grubb’s arguments in support of requiring basic care as the alternative to abandonment. Finally, we consider the dominant rationale for the basic care exception most utilized today, that of the rights and interests of others.

### A. Refusal of basic care as inconsistent with respect for human dignity

Basic care can be seen as activity that protects the dignity of the individual, but also more generally as an integral part of respect for human dignity *per se*. Since the enactment of the MCA, there has been a clear shift in the increasing recognition of the importance of the concept of human dignity in domestic jurisprudence concerning end of life decision-making. At a supranational level, recognition of the inalienable right to human dignity and guarantees to protect it can be found in instruments including the United Nations’ Universal Declaration of Human Rights, the Council of Europe’s Biomedicine Convention, and the Convention on the Rights of Persons with Disabilities.^116^ Whilst not explicitly mentioned in the European Convention on Human Rights, the European Court of Human Rights’s jurisprudence has identified it as the central premise upon which the convention is built.^117^ In England and Wales, references to human dignity abound within the case law relating to non-treatment.^118^ As Munby J recognized in *R (A*, *B*, *X*, *and Y) v East Sussex CC and the Disability Rights Commission (No 2)*, *‘*The recognition and protection of human dignity is one of the core values - in truth the core value - of our society.’^119^

Nevertheless, there is no consensus on what the protection of human dignity requires, and the caselaw demonstrates that dignity is being used both to protect the autonomy interests of individuals and to constrain those interests in the name of protecting the interests of third parties. Beyleveld and Brownsword offer two competing constructions of dignity, namely dignity as empowerment and dignity as constraint.^120^ Conceptualizing dignity as empowerment recognizes the manner in which it can be used to support choice, to reinforce autonomy.^121^ Raz wrote ‘Respecting human dignity entails treating humans as persons capable of planning and plotting their future. Thus, respecting people’s dignity includes respecting their autonomy, their right to control their future.’^122^ This conceptualization of human dignity was adopted by Hayden J in *North West London Clinical Commissioning Group v GU*^123^ in addressing the question of whether GU’s best interests required the continuation of clinically assisted nutrition and hydration. He stated that:

Whilst there is and can be no defining characteristic of human dignity, it is clear that respect for personal autonomy is afforded pre-eminence. Each case will be both situational and person specific…. Frequently, it will involve drilling down into the person’s life, considering what he or she may have said or written and a more general evaluation of the code and values by which they have lived their life.^124^

The European Court of Human Rights recognized the synthesis of dignity with autonomy in *Pretty*.^125^ Moreover, dignity is not restricted to those with capacity;^126^ the Council of Europe’s Parliamentary Assembly has emphasized that dignity requires account to be taken of previously expressed wishes and advance decisions.^127^ In *NHS Surrey Heartlands Integrated Care Board v JH*,^128^ Hayden J granted a declaration that there would be no liability for the consequences of not performing any invasive tests or treatment in accordance with JH’s valid advance decision. He emphasized that:

There is no obligation on a patient, who has decision-making capacity, to accept life-saving treatment. Doctors are not obliged to provide treatment and, perhaps more importantly, are not entitled to do so in the face of a patient’s resistance. This reflects a mature understanding of the importance of individual autonomy and respect for human dignity.^129^

However, dignity can also be conceptualized as constraint, as imposing limits upon personal choice. Waldron has argued that ‘dignity has to function as a normative idea: it is the idea of a certain status that ought to be accredited to all persons and taken seriously in the way they are ruled.’^130^ This construction of dignity emphasizes the value of human life and is objective in application, echoing Lord Hoffmann’s words in *Bland* ‘that there is an intrinsic value in human life, irrespective of whether it is valuable to the person concerned or indeed to anyone else.’^131^ This conception of dignity as constraint could be applied to require that a minimum level of care is provided to those unable to make decisions for themselves, including the offer of food and fluids and the provision of pain relief, on the basis that a person’s status as a human being requires this care to be provided.

In the words of Hayden J, the concept of human dignity advances ‘a universal understanding of a unique value intrinsic to the human condition.’^132^ However, whilst the concept can be seen as objective, it is subjective in its application, as Macdonald J recognized in *Tafida Raqeeb v Barts NHS Foundation Trust and others*, the content of human dignity in relation to a specific individual will be influenced by ‘the religious or cultural context’.^133^ Moreover, it is suggested that the more recent emphasis placed upon the patient’s own wishes and feelings,^134^ has further emphasized the subjective component of human dignity. As Hayden J recognized in *M v N*, the value of life is subjective, it should be considered from the point of view of the patient.^135^ He emphasized the importance of N’s known wishes in assessing whether the withdrawal of CANH would be in her best interests saying

There is an innate dignity in the life of a human being who is being cared for well, and who is free from pain. There will undoubtedly be people who for religious or cultural reasons or merely because it accords with the behavioural code by which they have lived their life prefer to, or think it morally right to, hold fast to life no matter how poor its quality or vestigial its nature. Their choice must be respected. But choice where rational, informed and un-coerced is the essence of autonomy. It follows that those who would not wish to live in this way must have their views respected too.^136^

Furthermore, Hayden J has emphasized that whilst good care and the environment in which that care is provided can promote human dignity,^137^ the challenge will be to recognize the point at which ‘treatment which may have enhanced the patient’s quality of life or provided some relief from pain … becomes futile, burdensome and inconsistent with human dignity.’^138^ Thus, although the guarantee of human dignity expresses respect for life, it will not always require the preservation of a specific life,^139^ particularly where it is clear that the individual concerned would not wish their life to be preserved.

Significantly, dignity does not merely require that all life be valued and protected; it also incorporates an element of equal treatment. It requires individuals to be valued in their own right, to be respected on the basis that they are human, without regard to characteristics such as whether they lack capacity. Both Jeremy Waldren and James Whitman have argued that dignity has occasioned a ‘levelling up’, requiring that the degree of respect and level of treatment previously only accorded to nobility be afforded to all persons.^140^ In the context of treatment and care refusal, this would suggest that those seeking to refuse treatment or care in anticipation of incapacity should not be subject to additional requirements, or restrictions, not imposed upon contemporaneous choices. However, as discussed above, the MCA attempts to secure a balance between empowering those who lack capacity and protecting the vulnerable, imposing form requirements upon advance decisions to refuse life-sustaining treatment. In *P v Cheshire West*, Lady Hale recognized that some restrictions might be necessary,^141^ but it is suggested that the exclusion of basic care from the ambit of a valid advance decision, a restriction not imposed upon contemporaneous refusals, cannot be justified solely on the basis of P’s lack of capacity.

There are multiple definitions of dignity, and in the case of basic care, a construction of dignity as constraint (emphasizing the interests of the community of which P is a part) appears to be being adopted, rather than an individualistic construction that promotes dignity as empowerment, reinforcing the individual’s right to make decisions for themself. This communitarian construction of dignity and the diminished importance accorded to autonomy is out of line with public policy in the twenty-first century. It can be argued that it is incompatible with the concept of human dignity and the principle of autonomy to suggest that an individual’s choices should be limited by the interests of others in a situation where the harm to the patient of ignoring their choice is arguably disproportionately higher than the harms to which others are exposed by the patient refusing basic care. At the time Grubb was writing, the jurisprudence relating to human dignity was in its infancy and the Human Rights Act 1998 had not been passed. However, the courts now consider both human dignity and human rights in determining how an individual lacking capacity should be treated. We suggest that in the same way that the refusal of basic care by an adult with capacity is respected, the protection of human dignity demands that the same respect be shown to advance decisions refusing care made in anticipation of incapacity. Nevertheless, countervailing dignity interests may also arise in relation to the issue of the rights and freedom of others; we consider this in Section V.C below.

### B. Recognition of a refusal of basic care as abandonment of the patient

Grubb suggests that respect for an individual’s refusal of basic care could be categorized as abandonment.^142^ In such a context, autonomy is trumped by the bioethical concept of non-maleficence; public policy demands that individual autonomy is constrained.^143^ Undoubtedly, a healthcare professional owes a duty of care to a patient in their care. *R v Instan* demonstrates that liability in criminal law can be imposed for a failure to provide care to another.^144^ However, where the individual has refused such care (albeit in advance), it would appear difficult to argue that there is a duty to rescue the individual from what are the inevitable results of their own autonomous decision.

Even if Grubb were correct at the time, in suggesting that a failure to provide basic care in accordance with the patient’s wishes would constitute abandonment, we would suggest that today that is no longer the case. There is today a long line of authority^145^ demonstrating that public policy now is clearly directed towards facilitating and respecting patient choice by those with mental capacity regarding refusal of treatment in all areas of healthcare, ostensibly at least even in the case of pregnant persons.^146^ In *PH v Betsi Cadwaladr University Health Board*,^147^ Hayden J refused to use the inherent jurisdiction to override PH’s contemporaneous refusal of feeding, stating, ‘The court has no business in telling capacitious individuals what is in their best interests nor any locus from which to compel others to bend to the will either of what capacitious individuals may want or what the court might consider they require.’^148^ We suggest that the same principle must apply to advance choices to refuse even basic care, to suggest otherwise is incompatible with the ethos of the MCA.

The patient-centred ethos adopted in relation to contemporaneous decision-making appears less applicable to individuals making anticipatory decisions who are framed as in need of protection from decisions that they may later regret.^149^ This same paternalistic concern may be applied to advance decisions due to the temporal and psychological distance between the decision being made and its later implementation. For that reason, as Hayden J stressed in *NHS Cumbria CCG v Rushton:*

[The statutory provisions and Codes of Practice] provide essential safeguards. They are [intended] to strike a balance between giving proper respect and recognition to the autonomy of a competent adult and identifying the risk that a person might find himself locked into an advance refusal which he or she might wish to resile from but can no longer do so. The balance is pivoted on the emphasis, in the case of life-sustaining treatment, given to compliance with the form specified by statute and codes.^150^

It is unclear why a refusal of basic care that is compliant with the enhanced requirements imposed upon advance decisions to refuse life-sustaining treatment should be devoid of legal effect.

### C. Refusal of basic care as a violation of the rights and interests of others

The third key argument—and today we would suggest the stronger of the two main arguments originally advanced by Grubb—relates to the rights and interests of others such as health professionals, other patients and family members, who may be affected by the individual’s refusal of basic care. This is seen as a separate justification flowing from a Millian utilitarian perspective, namely that an individual can choose freely, so long as those choices do not harm others.^151^ It recognizes that hospital patients will generally not be cared for in isolation, they may be situated in a busy ward or corridor, and consequently, that their choices impact upon others: healthcare professionals, visitors and other patients. Dying in such situations may not, therefore, be seen as a wholly private matter. Secondly, while respect for an individual’s advance decision can be seen as respecting their fundamental human rights to autonomy under Article 8 of the ECHR, that is qualified by reference to public health considerations and the rights and freedoms of others, Article 8(2).

If a hospital patient refuses all basic care, including hygiene measures, this could pose a potential risk of serious infection to other patients and healthcare professionals. It could be argued that in such a situation, the decision not to go along with the patient’s advance refusal may be justified under Article 8(2) in the light of the protection of health, or rights and freedoms of others. One possibility might be to place the patient refusing care in a private room, however, the viability of such a solution would depend upon the available space and staffing resources in the hospital at that time. The issue of resource allocation in relation to healthcare is an area where a wide margin of appreciation is left to member states,^152^ but we suggest that in the challenging context of NHS provision, the ability to protect human dignity (both at the individual and the community levels) may be constrained by the mechanics and context of delivery.

If resources were not a problem, could the refusal of basic care still be overridden on the basis that the refusal constituted a violation of the dignity of third parties? As Christian Starck explains, ‘Human dignity does not mean unlimited self-determination, but self-determination which is exercised on the basis that everyone—not simply the person claiming the right to self-determination—is of value in his or her own right.’^153^ Thus, account must be taken of the impact of one’s choices upon others.

The need to achieve a balance between the individual and healthcare professional’s (dignity) interests was recognized in *R (A, B, X and Y) v East Sussex CC and the Disability Rights Commission (No 2)*,^154^ which involved two sisters with considerably impaired mobility who wished to be lifted manually in certain situations, and the concerns of carers that manual handling posed a risk to their own physical health. Munby J recognized that dignity interests form the core of both Article 8 and Article 3 ECHR.^155^ Nevertheless, he emphasized that A and B’s rights ‘necessarily take effect subject to the corresponding rights of their carers,’^156^ that a balancing exercise must be undertaken.^157^ He rejected suggestions that undertaking the risks involved in manual handling is part of the role, or even something that carers are paid to do. Instead, he found that

‘Civil society has to balance the dignity of the patient against the dignity of the carer and can only do so by reference to the standards of the average human being. … Civil society cannot expect of its nurses, care assistants and others in similar positions, the abnegation and self-sacrifice of a Mother Teresa.’^158^

Moreover, the impact that the patient’s choices might have on family and friends, and healthcare professionals must be considered, choices that may occasion significant distress. In relation to relatives, the courts have been clear that although non-treatment decisions can be very challenging for close relatives, their views are not determinative; rather, the question of whether treatment should be withdrawn is a matter to be determined on the basis of the best interests of the individual themself.^159^ Significantly more attention has been paid by judges to the impact of non-treatment upon health care professionals, particularly nursing staff. In *Bland*, when considering the consequences of the removal of artificial nutrition and hydration from Tony Bland, Lord Mustill recognized that:

Whereas for almost all concerned the [withdrawal of CANH] will be a merciful relief, this will not be so for the nursing staff, who will be called on to act in a way which must be contrary to all their instincts, training and traditions. … The interval between the initiation of the proposed conduct and the death of Anthony Bland will be a very stressful period.^160^

Lord Goff recognized that it would be appropriate to give Bland a sedative to suppress symptoms that might distress his nurses or family following the discontinuance of artificial feeding.^161^ However, despite recognizing the negative impact the removal of CANH would have upon others, the Law Lords held that Bland’s best interests required that treatment be discontinued.^162^ Similarly, in the later case of Ms *B v An NHS Hospital Trust*,^163^ Dame Butler Sloss accepted that the treating clinicians were very distressed by the prospect of withdrawing ventilation in accordance with Ms B’s refusal of consent to continued ventilation because they felt that such an action would be tantamount to killing her. Nevertheless, Dame Butler-Sloss emphasized that as an adult with capacity, Ms B had the right to refuse even life-sustaining treatment and that treatment in the face of that refusal was unlawful. She stressed that ‘if the doctors are for any reason unable to carry out the wishes of the patient, their duty is to find other doctors who will do so.’^164^ In the more recent case of *GU* Hayden J held that

The guidance emphasises that the central point to keep in mind is that the decision making process is about the best interests of the individual patient not what is best for those who are close to, or around them. I was told …that the discontinuance of life sustaining treatment in the kind of circumstances arising here causes distress to staff, other patients and their families. … I have no doubt that these cases cause deep distress to others in the hospital. … I have equally no doubt that these considerations have no place at all in evaluating GU's best interests. Factoring these matters into the decision process is both poor practice and ethically misconceived^165^

However, the following year in *Barts Health NHS Trust v Dance & others (Re Archie Battersbee*^166^ he held that an individual’s best interests encompassed consideration of the distress experienced by healthcare providers in caring for the child. Hayden J emphasized the relevance of the nurses’ distress at continuing to provide intensive support to a child, knowing that this could only prolong death,

It is crucial that I survey the entire canvas of the available evidence, the reaction of the nurses, as reported to me, is a part of that canvas, particularly when considering what weight should be afforded to respect for Archie’s dignity and autonomy.^167^

Patients and clinicians may disagree about what will constitute the most appropriate treatment and care, but a patient cannot simply demand a particular treatment; the availability of any treatment will depend upon it being judged clinically appropriate.^168^ However, as these cases demonstrate, disagreements may be based upon the broader interests of healthcare professionals, not merely clinical judgment. While we appreciate how difficult and distressing these situations can be for individuals, we suggest that the values and personal interests of healthcare professionals should not undermine an individual’s capacitous refusal, or determine their best interests. Care must remain patient-, not professional-centred. For that reason, we would also reject the availability of a right to conscientious objection in this context.^169^ Given the need for consent to render care or treatment legally innocuous, it would be entirely unacceptable to permit a healthcare professional to treat a patient in the face of their capacitous refusal (anticipatory, or contemporaneous) on the basis of their own values. Such a claim is unlikely to be successfully supported by reference to Article 9 of the ECHR, not least given the qualified nature of that right.^170^ Where the clinician feels unable to support the patient’s decision, professional practice guidance provides discretion to clinicians not to support a patient’s choice. In such a case, there is a duty to transfer care to another practitioner.^171^

## VI. CONCLUSIONS

Nearly 30 years after the Law Commission deliberations on mental incapacity began, the approach taken to the refusal of basic care in the context of advance decisions remains an instance where respect for autonomy is circumscribed by public policy. As detailed above, this is a context where respect for human dignity is all too often constructed as a constraint rather than empowerment. In a field of law that has adopted autonomy as the golden principle, this approach is inconsistent with the ethos underpinning the MCA and diminishes the potential for individuals to take decisions for themselves. While the 2005 Act itself did not incorporate an exclusion of basic care, the Code published in 2007 remains unrevised.

The Code’s exclusion of basic care purports to diminish the right to exercise precedent autonomy set out in the Act; it endorses medical paternalism without seeking to explain the rationale for the exception, save for quoting public policy reasons. There is no evaluation of the proportionality of the exclusion, nor engagement with other public policy rationales that would suggest that the default position should be that individuals may refuse any treatment, or care, in line with their own values. The elusive definition of basic care aside, this discrepancy between the Act and the Code has led to a lack of clarity both in terms of intention and implementation. At the drafting stage, it is questionable to what extent those drawing up an advance decision (including their lawyers if involved) engage with, or are even aware of the exclusion of basic care set out in the Code. If the action refused in an advance decision is deemed basic care, it is likely that that element of their advance decision will be treated as non-binding, it will not be given effect as if the individual had made it at the time when the question arises.^172^ Moreover, even though the MCA requires consideration of the individual’s ‘past and present wishes and feelings (*and, in particular, any relevant written statement made by him when he had capacity*),’^173^ as part of the best interests assessment, it is difficult to imagine significant weight being given to the person’s refusal of basic care, given the exclusion set out in the Code. Thus, the individual’s precedent autonomy is thwarted.

The lack of clarity also puts clinicians faced with an advance refusal of basic care in an extremely difficult position. To the extent that the Code repeats the legislation, it is binding; compliance is required of all concerned. However, the basic care exclusion is not part of the MCA, and therefore, we argue that it is not legally binding. The Code constitutes guidance, is advisory in nature, not obligatory, despite the fact that the core exception is repeated at the level of individual Trust policy, in professional guidance, and indeed in guidance produced by third sector organizations. Therefore, faced with a refusal of basic care, the clinician is not bound to disregard it. However, the Code provides a clear statement that basic care cannot be refused, and the GMC clearly expresses an expectation that basic care will be provided to all patients in its guidance:

If a patient is not receiving basic care to meet their needs, you must act to make sure the patient is cared for as soon as possible, for example by asking someone who delivers basic care to attend to the patient straight away.^174^

Significantly, this guidance does not consider that the patient may have refused basic care, assuming that all patients should receive basic care. It is well accepted that patients with capacity can refuse basic care;^175^ we suggest that the same principle should apply to advance refusals. Faced with an advance refusal of basic care, the clinician has three options. They could determine that the advance decision is not valid and provide basic care without incurring liability provided that that is consistent with the individual’s best interests; withhold basic care on the basis of a reasonable belief that the advance decision is valid and applicable; or provide basic care pending a declaration from the Court of Protection about whether the advance refusal of basic care is valid.^176^ The Act is heavily weighted in favour of clinical discretion,^177^ but this state of affairs is untenable. Ultimately, if those considered choices set out in an advance decision are to be overridden this should only be under provisions inserted into the primary legislation with appropriate procedural safeguards, including a requirement that this matter be referred for determination to the Court of Protection and that the decision of the Court should be a matter for a fully reported decision.

The purported blanket ban on an advance refusal of basic care is simply too blunt a tool; the time has come to re-evaluate the basic care exception. One of the inevitable challenges in interpreting and implementing advance decisions is in the way in which clinical discretion is built into the legislation itself through the requirement that the clinician determine whether the advance decision is valid and applicable, subject to confirmation by the Court of Protection as required. In relation to basic care, the Code of Practice adds a further layer to this application of discretion. These are issues that will need to be considered in the future in relation to reform of the law in this area.

We fully acknowledge that public policy considerations arise, but we suggest that a more nuanced approach is required. Where an individual forms a well-considered and informed decision to refuse elements of, or even all, basic care, we suggest that human dignity and autonomy would normally require that such decisions are determinative, regardless of whether made contemporaneously, or in advance, it should not be a matter of what a third party deems appropriate in the circumstances. In recognition of the concerns raised by a refusal of basic care, we suggest that the more stringent requirements of advance refusals of life-sustaining treatment be applied, requiring refusals to be signed, written and witnessed.^178^ Moreover, wishes and values statements can be extremely helpful in providing the context for an advance refusal, this will be particularly so in the case of an advance refusal of basic care. Such a narrative would demonstrate that the individual had fully understood the consequences of their decision and would, for example, express their decision that they would prefer to refuse pain relief in the hope that they might experience even short periods of lucidity with their family, despite the suffering that would entail. In the context of dementia, it would allow an individual to explain their refusal of food and fluid from the point where they no longer recognized their spouse as a deliberate decision to stop eating, and therefore die, at this stage of the debilitating disease, rather than continue to live. This would represent a useful tool for the clinician charged with implementing the refusal to satisfy themself of its validity and applicability and to allay fears that the patient is being abandoned. It would ensure that the patient’s autonomy and dignity could be promoted; that treatment and care centred upon their wishes could be provided. If refusals of basic care are to be excluded, the reasons why such an advance refusal would not be seen as valid and applicable should be clearly stated and documented.

There are clear asymmetries between anticipatory and contemporaneous decision-making, yet it remains the case that currently a clear, signed and witnessed advance decision excluding basic care could be overridden. Despite the increased emphasis over the last 30 years on autonomy and human dignity in this area, the position of basic care remains an anomaly, a significant infringement hiding behind the cloak of underexplained ‘public policy considerations.’ A detailed consideration of the policy imperatives is urgently required, but this is not an issue that should be left to be resolved in the courtroom reacting to a crisis situation. Instead, this is a matter that should be addressed by policy makers interrogating these questions through a 21st century lens. The review of the Mental Capacity Act Code presents an ideal opportunity to reflect further on the legitimacy of the existing ‘basic care’ exclusion and to resolve the current anomaly.

## Footnotes

1 Homer *The Odyssey, XII, ‘Scylla and Charybdis’*, discussed in D Morgan, ‘Odysseus and the Binding Directive: Only a Cautionary Tale? (1994) 14 Legal Studies 411.

2 See eg, comments made by Hayden J in *Guys and St Thomas’ NHS Foundation Trust & South London and Maudsley NHS Foundation Trust v R* [2020] EWCOP 4, [57] and [67].

3
*Re T (Adult: Refusal of Treatment)* [1993] Fam 95; *Airedale NHS Trust v Bland* [1993] AC 789; *St George’s Healthcare NHS Trust v S* [1999] Fam 26; *Ms B v An NHS Hospital Trust* [2002] EWHC 429 (Fam); *R (on the application of Nicklinson and another) v Ministry of Justice* [2014] UKSC 38.

4 ss 24-26 MCA 2005.

5 Department for Constitutional Affairs, *Mental Capacity Act 2005. Code of Practice* (TSO 2007), para 9.28. We suggest that basic care provided by a healthcare professional could constitute a procedure within the meaning of ‘treatment’ as defined in s 64 MCA 2005 and therefore could be refused in an advance decision to refuse treatment under ss. 24-26 MCA 2005.

6 On the asymmetries between contemporaneous refusals and advance decisions, see eg S Michalowski, ‘Advance Refusals of Life-sustaining Medical Treatment: The Relativity of an Absolute Right’ (2005) 68 Modern Law Review 958; A Buchanan and D Brock, *Deciding for Others: The Ethics of Surrogate Decision-making* (CUP 1990) 103–7; S Halliday, ‘Legislating to Give Effect to Precedent Autonomy: Comparative Reflections on Legislative Incompetence’ (2011) 11 Medical Law International 127.

7 In the literature, ‘basic care’, ‘nursing care’, ‘comfort care’, and ‘essential care’ are used synonymously.

8 MCA COP 2007, para 9.28: ‘An advance decision cannot refuse actions that are needed to keep a person comfortable (sometimes called basic or essential care)…’

9 See Section IV.B below.

10 HM Government, *Draft Mental Capacity Act 2005 Code of Practice. Including the Liberty Protection Safeguards*, 2022, para 11:37.

11 For a recent example, see *NHS Surrey Heartlands Integrated Care Board v JH* [2023] EWCOP 2.

12
*Aintree University Hospitals NHS Foundation Trust v James* [2013] 3 WLR 1299; *Briggs v Briggs* [2016] EWCOP 53.

13 ibid.

14 Law Commission Consultation Paper No 129, *Mental Incapacitated Adults and Decision Making, Medical Treatment and Research*, 1993; Law Commission *Mental Incapacity Report*, Law Com No 231, 1995.

15 Pain relief is not included within the definition of basic care provided by the Code. However. GMC guidance in 2010 and BMA guidance in 2025 define basic care as including pain relief (GMC *Treatment and care towards the end of life: good practice in decision-making*, 2010, updated 2024, 50; BMA *Core Ethics Guidance*, 2025, 4.9). The NHS National End of Life Care Program Guide *Improving end of life care, Advance decisions to refuse treatment A guide for health and social care professionals* (2013) recognizes that some may categorize ‘drug measures to ensure comfort’ as basic care but notes that such measures can have harmful effects, 17. In contrast to the GMC and BMA guidance, the NHS guide recognises that individuals can refuse pain relief, but that the validity and applicability of such advance decisions ‘would require stringent scrutiny,’ 19. This suggests that a very high degree of capacity and specificity (both in terms of the drugs refused and the circumstances in which the refusal is to apply) would be required for an advance refusal of pain relief to be regarded as binding.

16 The MCA COP 2007 includes ‘the offer of food and water by mouth’ in its definition of basic care, para 9.28. Similarly, the GMC guidance states ‘an advance refusal of food and drink has no force,’ (n 15) 50. The professional guidance and the Code all explicitly exclude CANH from the definition of basic care, categorising it as treatment and therefore as something that can validly be refused in an advance decision, (BMA 2025 (n 15) 4.9; GMC 2010 (n 15) 50; MCA COP 2007, para 9.28).

17 cf Sir Thomas Bingham MR in *Bland* (n 3), suggesting that labels were unhelpful: ‘It does not, however, seem to me crucial whether this is regarded as medical treatment or not, since whether or not this is medical treatment, it forms part of the patient’s medical care and I cannot think the answer to this problem depends on fine definitional distinctions,’ 809.

18 Per Goff LJ, in *Collins v Willcock* (1984) 79 Cr App R 229, 234.

19 See further, Hoffmann LJ in *Bland* (CA) (n 3) 831, referring to the ‘ethical principle which requires doctors and hospitals to provide the patients in their care with such medical attention and nursing as they are reasonably able to give.’

20 In their seminal work on human dignity, Deryck Beyleveld and Roger Brownsword offer two constructions of dignity: dignity as empowerment and dignity as constraint, see D Beyleveld and R Brownsword, *Human Dignity in Bioethics and Biolaw* (OUP 2001). These constructions are used in this article as the most commonly used constructions of dignity in end of life decision making, see S Halliday, ‘Comparative Reflections upon the Assisted Dying Bill 2013: A Plea for a More European Approach’ (2013) 13 Medical Law International 135.

21 D Dudzinski and S Shannon, ‘Competent Patients’ Refusal of Nursing Care’ (2006) 13 Nursing Ethics 608, 609.

22 ibid.

23 H Aveyard, ‘The Patient Who Refuses Nursing Care’ (2004) 30 Journal of Medical Ethics 346, 346, citing T.H. Englehardt *Foundations of Bioethics* (OUP 1986).

24 See generally M Gunn, ‘Mental Incapacity: The Law Commission’s Report’ (1995) 7 Child and Family Law Quarterly 209; B Hale, ‘Mentally Incapacitated Adults and Decision-Making: The English Perspective’ (1997) 20 International Journal of Law and Psychiatry 59; P Wilson, ‘The Law Commission’s Report on Mental Incapacity: Medically Vulnerable Adults or Politically Vulnerable Law’ (1996) 4 Medical Law Review 227.

25 LC Rep (n 14).

26
*Re T* (n 3).

27 ibid 115.

28
*Bland* (n 3).

29 ibid 864.

30 ibid.

31 ibid.

32 BMA, *Information on Advance Directives* (BMA 1992); BMA, *Statement on Advance Directives* (BMA 1994); BMA, *Advance Statements About Medical Treatment*, *Code of Practice* (BMA 1995).

33 LC CP (n 14).

34 A Grubb, ‘Treatment Without Consent: Adult. *Re T (Adult: Refusal of Treatment)*’ (1993) 1 Medical Law Review 83.

35
*Re T* (n 3), 102. The extent to which a pregnant person may exercise precedent autonomy in relation to their care and treatment remains an acute source of controversy, but further consideration of this reservation goes beyond the scope of this article. For a detailed consideration of this issue, see S Halliday, *Autonomy and Pregnancy: A Comparative Analysis of Compelled Obstetric Intervention* (Routledge 2016) 40–93; S Fovargue, ‘In Whose Best Interests? Childbirth Choices and Other Health Decisions’ (2021) 137 Law Quarterly Review 604; S Halliday, ‘Pregnancy and Severe Mental Illness: Birth Choices, Best Interests and the Untapped Potential of Advance Decisions’ (2024) 24 Medical Law International 14.

36 See further, Hoffmann LJ in *Bland* (CA) (n 3) 831—‘another ethical principle … demands that we should show kindness and humanity to our fellow human beings. At the most basic level, this principle insists that we should, if we are able to do so, provide food and shelter to a human being in our care who is unable to provide them for himself. If someone allows a small child or invalid in his care to starve to death, we do not say that he allowed nature to take its course. We think he has committed a particularly wicked crime. We treat him as if he had introduced an external agency of death. It is the same ethical principle which requires doctors and hospitals to provide the patients in their care with such medical attention and nursing as they are reasonably able to give.’

37 Grubb (n 34), 85, our emphasis.

38 ibid.

39 ibid.

40 LC CP (n 14), para 3.26.

41 ibid, para 3.25.

42 I. Kennedy and A Grubb, ‘Withdrawal of Artificial Hydration and Nutrition: Incompetent Adult: *Airedale NHS Trust v. Bland*’ (1993) 1 Medical Law Review 359.

43
*Bland* (n 3), 809.

44 Kennedy and Grubb (n 42), 367.

45 ibid.

46 LC CP (n 14), 41.

47 LC Rep (n 14).

48 ibid, para 5.34.

49 ibid, para 5.34 and Draft Mental Incapacity Bill Clause 9(7)(a), (8).

50 ibid, para 5.34.

51 ibid.

52 ibid.

53 ibid.

54 Lord Chancellor’s Department, *Consultation Paper: Who Decides? Making Decisions on Behalf of Mentally Incapacitated Adults*, 1997.

55 ibid, paras 4.35–4.37.

56 ibid, para 4.37.

57 Lord Chancellor’s Department, *Making Decisions: The Government’s Proposals for Making Decisions on Behalf of Mentally Incapacitated Adults: A Report Issued in the Light of Responses to the Consultation Paper Who Decides?* 1999.

58 ibid, para 20.

59 See eg, comments made by Ann Winterton MP upon publication of the Green Paper, HC Deb 10 December 1997: Col 1018.

60 ‘BMA’s Death Code Attacked as Unethical’ (*The Times*, 12 August 1999) 14.

61 See eg, ‘End Backdoor Euthanasia Say Dissidents’ (*The Times*, 24 June 1999) 12; ‘Elderly Patients ‘Left Starving to Death in NHS’ (*The Daily Telegraph*, 6 December 1999) 1; ‘Charity Wants Inquiry into ‘Starving of Elderly’ (*The Independent*, 7 December 1999) 11; ‘Doctors Call for Deaths Inquiry’ (The Times, 7 December 1999) 8.

62 ‘Doctors Call for Deaths Inquiry’ (*The Times*, 7 December 1999) 8.

63 Upon sentencing Shipman on 31 January 2000, Forbes J said, ‘None of your victims realised that yours was not a healing touch. None of them knew that in truth you had brought her death, death which was disguised as the caring attention of a good doctor.’ Cited in Dame Janet Smith DBE, *The Shipman Inquiry: First Report*, Volume 1. ‘Death Disguised,’ 2002.

64 N 32.

65
*Making Decisions* (n 57), para 20.

66 See eg, Evidence from Department for Constitutional Affairs, Joint Committee on the Draft Mental Incapacity Bill—Minutes of Evidence Q742—744 explaining the importance of including advance refusals of treatment within the comprehensive statutory scheme, setting out their relationship to LPAs and the jurisdiction of the Court of Protection to determine their validity <https://publications.parliament.uk/pa/jt200203/jtselect/jtdmi/189/3102206.htm> accessed 29 July 2025.

67 Clauses 23–25.

68 This again reflected concerns regarding the allegations that withdrawal of treatment could constitute euthanasia. Joint Committee on the Draft Mental Incapacity Bill *Report: Draft Mental Incapacity Bill*, *2003*, HL Paper 189-I, HC 1083-I, 6.

69 ibid. See eg, responses to Q142–148 by C Evers (Alzheimer’s Society), S Foster (Mind), R Goss (Patient Concern), and K Willmington (Help the Aged, Making Decisions Alliance).

70 ibid Q268.

71 ibid Q265.

72 ibid, Joint memorandum by the Catholic Bishops’ Conference of England and Wales and the Linacre Centre for Healthcare Ethics (MIB 1001), paras 24–25. The Royal College of Psychiatrists went further still, see Memorandum from the Royal College of Psychiatrists (MIB 824) [3.10].

73 Report (n 68), para 201.

74 Report (n 68), Recommendation 64.

75 Government Response to Joint Committee’s Recommendations, R64, February 2004.

76 LC Rep (n 14).

77 ss 24–26 MCA 2005.

78 s 24(1) MCA 2005.

79 s 26(2) MCA 2005. Note the COP and the GMC guidance emphasise that a doctor should presume P had the requisite capacity unless there are grounds for doubt, *MCA MCA COP*, 2007, paras 9.39 and 9.8; GMC 2010 (n 15), para.s 70(b) and 72.

80 s 24(1) MCA 2005. In *NHS Cumbria CCG* v *Rushton* [2018] EWCOP 41, Hayden J emphasized the need for ‘a clear statement of the decision, the treatment to be refused and the circumstances in which the decision will apply,’ [16].

81 s 25(5)(6) MCA 2005.

82 s 26(1) MCA 2005.

83 s 25(2)(a) MCA 2005.

84 s 25(2)(b) MCA 2005.

85 s 25(2)(c) MCA 2005, see for example *Re PW (Jehovah’s Witness: Validity of Advance Decision)* [2021] EWCOP 52.

86 S.25(4)(c) MCA 2005.

87
*R (on the application of Burke) v GMC* [2005] QB 424.

88 s 62 MCA 2005; cf s 4(5) MCA 2005.

89 s 4(6)(a) MCA 2005. Advance decisions are limited to treatment refusals and therefore a refusal of social care, such as admission to a care home, would not be binding, but would similarly be a factor to be considered within the best-interests assessment in determining where P should live.

90
*Burke* (n 87).

91 See eg, comments made by Baroness Ashton of Upholland (the Parliamentary Under-Secretary of State, Department for Constitutional Affairs) during the Committee stage, HL Deb 01 February 2005 vol 669 col 125 and 126. Cf Irish Assisted Decision-Making (Capacity) Act 2015, incorporating a provision excluding basic care and providing a definition thereof, s 85.

92 MCA COP 2007, foreword.

93 MCA COP 2007, 1.

94 ibid, Introduction, 1–2.

95 [2018] UKSC 46.

96 MCA COP 2007, 6.18.

97
*Y* (n 95) [97].

98 ibid [107].

99 See further per Hayden J *Rushton* (n 80) [25].

100 s 4(6)(a) MCA 2005.

101 J Laing, ‘A Lethal Power’ (2012) 162 New Law Journal 1444 and *More Care: Less Pathway A Review of Liverpool Care Pathway for Dying Patients* (Department of Health 2013).

102 LCP Review (n 101) 3.

103 ibid 3–4.

104 NICE Guidance [NG 31], 2015 <https://www.nice.org.uk/guidance/ng31> accessed 29 July 2025.

105
*Y* n 95 [77]. Whilst the MCA COP 2007 provides that ‘Healthcare and social care staff should also refer to relevant professional guidance when making decisions regarding life-sustaining treatment’ (para 5.31), no similar statement is found in relation to advance decisions.

106 See (n 15) and (n 16).

107 GMC 2010 (n 15), 50; BMA 2025 (n 15), 4.9.

108 BMA 2025 n 15, 4.9; *Burke* (n 87).

109 BMA 2025 (n 15), 4.9. The DOH and Social Care, *Reference Guide to Consent for Examination or Treatment* (2nd edn, DOH and Social Care 2009) also includes ‘actions to keep a person … free from distress’ in its definition of basic care, 21 [51]. Note this guidance is still current, but predates *Montgomery v Lanarkshire Health Board* [2015] UKSC 11.

110 Independent Inquiry into care provided by Mid Staffordshire NHS Foundation Trust January 2005—March 2009 Volume I (2010) <https://assets.publishing.service.gov.uk/government/uploads/system/uploads/attachment_data/file/279115/0898_i.pdf> accessed 29 July 2025.

111 J Laing, ‘A Lethal Power’ (n 101) and *More Care: Less Pathway A Review of Liverpool Care Pathway for Dying Patients* (Department of Health 2013).

112 See Hoffmann LJ, *Bland* (n 3), 831.

113 para 9.28.

114 N 16.

115 A notable example is Tony Nicklinson, who challenged the current legal prohibitions of assisted dying. As he had capacity, he was able to refuse all nutrition and fluids, see Lord Neuberger in *Nicklinson* (n 3) [6].

116 UN Universal Declaration of Human Rights, adopted by the UN General Assembly, Paris, 10/12/1948, art 1; Council of Europe, Convention on Human Rights and Biomedicine, Oviedo, 4/4/1997, art 1; UN CRPD, adopted 12/12/2006, art 3(a).

117 See eg *Pretty v UK* (2002) 35 EHRR 1, [65] and *Svinarenko and Slyadnev* (App no 32541/08, [2014] ECHR 973), [138].

118 See eg, *M v N* [2015] EWCOP 76; *Alder Hey Children’s NHS Foundation Trust v Evans & Anor* [2018] EWHC 308 (Fam); *North West London CCG v GU* [2021] EWCOP 59; and *Guy’s and St Thomas’ NHS Foundation Trust v A & Ors* [2022] EWHC 2422.

119 [2003] EWHC 167 (Admin), (2003) 6 CCLR 194, [86].

120 D Beyleveld and R Brownsword, *Human Dignity in Bioethics and Biolaw* (OUP 2001).

121 This approach to dignity has been adopted by the German Constitutional court holding that the ability to make decisions about how our lives end is fundamental to our very being, that human dignity demands that respect for individuals and their personal values can only be protected if individuals can choose for themselves how their life ends. BVerfG, Urteil des Zweiten Senats vom 26. Februar 2020—2 BvR 2347/15, 211.

122 J Raz, *The Authority of Law: Essays on Law and Morality* (2nd edn, OUP 2009), 221.

123 [2021] EWCOP 59.

124 ibid [64].

125
*Pretty v United Kingdom* (n 117), [65]; see also *Hass v Switzerland* [2011] ECHR 2422, [51].

126 As Lord Hoffmann recognized in *Bland* (n 3), ‘It is demeaning to the human spirit to say that, being unconscious, he can have no interest in his personal privacy and dignity, in how he lives or dies,’ 829. See also Munby J in *Burke* (n 87), [58].

127 Parliamentary Assembly, ‘Protecting Human Rights and Dignity by Taking into Account Previously Expressed Wishes of Patients’ Resolution 1859 (2012) <https://pace.coe.int/en/files/18063#trace-4> accessed 29 July 2025.

128 N 11.

129 ibid [9].

130 J. Waldron, ‘How Law Protects Dignity’ (2012) 71 Cambridge Law Journal 200, 202.

131
*Bland* (n 3), 826.

132
*North West London CCG v GU* (n 118), [86].

133 [2019] EWHC 2530 (Fam), [176]. In *Guy’s and St Thomas’ Children’s NHS Foundation Trust v Pippa Knight* [2021] EWHC 25 (Fam) Poole J stated, ‘given the very different ideas expressed to the court about what would constitute dignity for Pippa in life and in her dying, I shall not presume to adopt some supposedly objective concept of dignity to determine her best interests,’ [86].

134
*Aintree* (n 12), [39].

135 [2015] EWCOP 76.

136 ibid [72].

137 See eg, *Alder Hey Children’s NHS Foundation Trust* v *Evans & Anor* [2018] EWHC 308 (Fam), [54–56].

138
*GU* (n 118), [105]; see also *Evans* (n 137), [56].

139 In *Evans* (n 137), dignity required that life-sustaining treatment be withheld; in *Raqeeb* (n 133), it required that such treatment be continued.

140 See eg, J Waldron, ‘Dignity and Rank: In Memory of Gregory Vlastos’ (2007) 2 Archives Européennes de Sociologie 201, 215ff; J Whitman, *Harsh Justice: Criminal Punishment and the Widening Divide Between America and Europe* (OUP 2003).

141 [2014] UKSC 19, [45].

142 N 37. This was the argument advanced by James Munby QC (later Munby LJ) when acting as counsel in *Bland*, namely—that the doctor owed a duty of care that would not permit the withdrawal of food, rather than treatment, (n 3), 836.

143 For further discussion of the concepts of autonomy and non-maleficence as concepts in biomedical ethics, see T Beauchamp, JF Allen and J Childress, *Principles of Biomedical Ethics* (8th edn, OUP 2019).

144 [1893] 1 QB 450, 453–54. Instan had failed to provide her aunt, with whom she lived, with food. Whilst the aunt’s death was attributed to gangrene, Instan was convicted of manslaughter, her failure to give food to her aunt being deemed to have accelerated her death.

145
*Re T* (n 3); *Re B* (n 3); see further P Singer ‘Ms B and Diane Pretty: a Commentary’ (2002) 28 Journal of Medical Ethics 234; *Aintree* (n 12); *Briggs v Briggs* (n 12).

146
*Re MB (Medical Treatment)* [1997] 2 FLR 426 and *St George’s* (n 3). See further (n 35).

147 [2022] EWCOP 16.

148 ibid [19].

149 For specific consideration of this in relation to the obstetric context, see eg *Guys and St Thomas’ NHS Foundation Trust (GSTT) and South London and Maudsley NHS Foundation Trust (SLAM) v R* [2020] EWCOP 4, [65]; Halliday (2024) (n 35).

150
*Rushton* (n 80), [22].

151 JS Mill, *On Liberty*, first published 1859 (OUP 1985).

152 See eg discussion in *University College London Hospitals NHS Foundation Trust v MB* [2020] 882 (QB).

153 C Starck, ‘The Religious and Philosophical Background of Human Dignity and its Place in Modern Constitutions’ in D Kretzmer and E Klein (eds), *The Concept of Human Dignity in Human Rights Discourse* (Kluwer Law International 2002) 179, 181. See also *Lopes-Ostra v Spain* (1994) 20 EHRR 277, [51].

154 N 119.

155 ibid [93].

156 ibid [116].

157 ibid [129].

158 ibid [149].

159 See eg, *Barts Health NHS Trust v Dance & others (Re Archie Battersbee)* [2022] EWHC 80.

160
*Bland* (n 3), 898.

161
*Bland* (n 3), 870.

162 BMA 1995 (n 32), 5.1.

163 [2002] EWHC 429 (Fam).

164 ibid [100].

165 N 118, 100.

166 N 159.

167 ibid [24].

168
*Burke* (n 87).

169 There are two statutory exceptions that protect the right to conscientious objection in England—s 4 of the Abortion Act 1967 and s 38 of the Human Fertilisation and Embryology Act 1990 (relating to reproductive technology treatment and embryo research).

170 See further admissibility hearing in *Pichon and Sajous v France*, Application no 49853/99 (ECtHR, 2 October 2001) discussed in A. Lamačková, ‘Conscientious Objection in Reproductive Health Care: Analysis of *Pichon and Sajous v. France*’ (2008) 15 European Journal of Health Law 7; M. Campbell ‘Conscientious objection, health care and Article 9 of the European Convention on Human Rights’ (2011) 11 Medical Law International 284; J. V. McHale ‘Health Care Choices, Faith and Belief in the Light of the Human Rights Act 1998: New Hope or Missed Opportunity?’ (2008) 9 Medical Law International 279; *Grimmark v Sweden*, Application no 43726/17 (ECtHR, 11 February 2020); *Steen v Sweden*, Application no 62309/17 (ECtHR, 11 February 2020); W. Brzozowski 'The Midwife’s Tale: Conscientious Objection to Abortion after *Grimmark* and *Steen*' (2021) 10 Journal of Law and Religion 298.

171 See also, LC Rep No 231 (n 14), para 5.28; General Medical Council, *Personal Beliefs and Medical Practice* (General Medical Council 2013); *Ms B* (n 3).

172 cf s 26(1) MCA 2005.

173 s 4(6)(a) MCA 2005.

174 GMC *Good Medical Practice*, 2024, [75a].

175 Refusals of basic care may raise safeguarding concerns, but if the patient has capacity to refuse care (including food and fluids), the most that a clinician can do is encourage the patient to accept care. There can be no suggestion of forcibly feeding or washing the patient.

176 s 26 MCA 2005.

177 s 26(2) MCA 2005.

178 It is worth noting that the Code’s exclusion of basic care is set out under the subtitle ‘What rules apply to advance decisions to refuse life-sustaining treatment.’

